# The KDM Inhibitor GSKJ4 Triggers CREB Downregulation via a Protein Kinase A and Proteasome-Dependent Mechanism in Human Acute Myeloid Leukemia Cells

**DOI:** 10.3389/fonc.2020.00799

**Published:** 2020-06-05

**Authors:** Michela Illiano, Mariarosaria Conte, Alessia Salzillo, Angela Ragone, Annamaria Spina, Angela Nebbioso, Lucia Altucci, Luigi Sapio, Silvio Naviglio

**Affiliations:** Department of Precision Medicine, University of Campania Luigi Vanvitelli, Naples, Italy

**Keywords:** GSKJ4, acute myeloid leukemia (AML), cAMP response element-binding protein (CREB) downregulation, proteasome-mediated degradation, protein kinase A (PKA)

## Abstract

Acute myeloid leukemia (AML) is a progressive hematopoietic-derived cancer arising from stepwise genetic mutations of the myeloid lineage. cAMP response element-binding protein (CREB) is a nuclear transcription factor, which plays a key role in the multistep process of leukemogenesis, thus emerging as an attractive potential drug target for AML treatment. Since epigenetic dysregulations, such as DNA methylation, histone modifications, as well as chromatin remodeling, are a frequent occurrence in AML, an increasing and selective number of epi-drugs are emerging as encouraging therapeutic agents. Here, we demonstrate that the histone lysine demethylases (KDMs) JMJD3/UTX inhibitor GSKJ4 results in both proliferation decrease and CREB protein downregulation in AML cells. We found that GSKJ4 clearly decreases CREB protein, but not CREB mRNA levels. By cycloheximide assay, we provide evidence that GSKJ4 reduces CREB protein stability; moreover, proteasome inhibition largely counteracts the GSKJ4-induced CREB downregulation. Very interestingly, a rapid CREB phosphorylation at the Ser133 residue precedes CREB protein decrease in response to GSKJ4 treatment. In addition, protein kinase A (PKA) inhibition, but not extracellular signal-regulated kinase (ERK)1/2 inhibition, almost completely prevents both GSKJ4-induced p-Ser133-CREB phosphorylation and CREB protein downregulation. Overall, our study enforces the evidence regarding CREB as a potential druggable target, identifies the small epigenetic molecule GSKJ4 as an “inhibitor” of CREB, and encourages the design of future GSKJ4-based studies for the development of innovative approaches for AML therapy.

## Introduction

Acute myeloid leukemia (AML) is a very hostile malignant disease deriving from a rapid clonal expansion of immature granulocyte precursors. As a consequence of multiple transcriptional alterations, due to chromosomal abnormalities, somatic mutations, and epigenetic variations, biological and clinical AML degrees are characterized by heterogeneity and unpredictable treatment responses (1). With an overall 5-year survival of <40%, AML is considered one of the deadliest leukemia subtypes. Current treatments, which include intensive chemotherapy rounds and bone marrow transplantation, are presumed to be only partially effective due to disease evolution and recurrent drug resistance achievement (2). Moreover, AML therapy is generally associated with a wide variety of side effects, including long-term complications and mortality (3). For this reason, the development of novel and more incisive therapeutic approaches with low toxicity are urgently demanded.

Normal hematopoiesis is mainly dependent on the controlled expression of critical genes by transcription factors. Notably, altered functions, by mutation and/or dysregulated expression, of these transcription factors play a relevant role in leukemogenesis (4). Speaking of which, cAMP response element-binding protein (CREB) is considered one of the most strictly related AML transcription factors (5).

As a member of a structurally related transcription factor family, which also includes activation transcription factor (ATF) and cAMP response element modulator (CREM), CREB proteins specifically recognize and bind DNA to cAMP-responsive element (CRE) promoter sites activating the transcription of specific target genes, including those affecting cell proliferation and survival. In response to various stimuli, such as hormones and growth factors, CREB leucine zipper domains are employed to generate dimeric homodimers and/or heterodimers, when combined with different proteins, complexes that precisely link deoxyribonucleic acid. Homodimer and heterodimer ratio undergoes continuous fluctuations into the cells in order to correlate signals from different pathways and to regulate the CREB transcriptional activity (6). Upon activation by phosphorylation at Ser133 by kinases, such as protein kinase A (PKA) and extracellular signal-regulated kinase (ERK)1/2, the co-activator CREB-binding protein (CBP) is engaged by CREB protein, and the CREB/CBP complex enhances the CREB transcriptional activity, facilitating the expression of the putative CREB-driven genes (7).

Previous studies have described CREB as a key regulator capable of driving both cell growth and survival in AML (8, 9). Specifically, CREB is generally overexpressed in AML cells causing growth rate increase and apoptosis resistance. Consistently, CREB knockdown induces decreased survival and proliferation in different leukemia cell lines (10). Conversely, an increased CREB expression has been associated with a reduced event-free survival in AML patients (11).

Based on the above findings, it is not surprising that CREB is becoming very attractive as a potential drug target for AML (4, 5, 12). Besides, increasing evidence indicates that dysregulation of histone modifications is widely involved in AML (13).

Epigenetic drugs are chemical and well-characterized compounds targeting disordered remodeling enzymes, thus modifying the chromatin cell state and changing the relative expression profile. Recently, several new epigenetic drugs have been developed, and some of them are standing out for their preclinical beneficial effects against cancer, including leukemia (14, 15). In this regard, GSKJ4 compound can be identified as a new inhibitor of the histone lysine demethylases (KDM) JMJD3 and UTX, showing a marked antiproliferative activity in different cancer types and, in particular, in AML cells (16–25). Here, we speculate that GSKJ4 treatment of AML cells could affect CREB pathway *via* a PKA and proteasome-dependent mechanism. The current investigation has been designed with the aim of defining the possible GSKJ4-mediated effects on CREB expression and function and the underlying molecular mechanisms in AML cells.

## Materials and Methods

### Chemical Reagents and Antibodies

Chemical reagents included bovine serum albumin (BSA) (Sigma-Aldrich, B2518), 3-(4,5-dimethylthiazol-2-yl)-2,5-diphenyltetrazolium bromide (MTT) (Sigma-Aldrich, M5655), trypan blue (Sigma-Aldrich, T6146), propidium iodide (PI) (Sigma-Aldrich, P4864), GSKJ4 (Sigma-Aldrich, SML0101), PD98059 (Sigma-Aldrich, P215), PKF118-310 (Sigma-Aldrich, K4394), MG132 (Alexis 133407-82-6), and H89 (Sigma-Aldrich, #B1427). Antibodies obtained from Santa Cruz Biotechnology: anti-nuclear factor kappa-light-chain-enhancer of activated B cells (NF-κB) p65(A) (sc-109), anti-Ub (P4D1) (sc-8017), anti-α-tubulin (B-7) (sc-5286). Antibodies purchased from Cell Signaling Technology: anti-CREB (#9198S), anti-p44/42 mitogen-activated protein kinase (MAPK) (ERK1/2) (#9102), anti-p-CREB (Ser133, #9198), anti-phospho-p44/42 MAPK (ERK1/2) (Thr202/Tyr204) (#9101). Anti-vinculin (ab13007) and anti-H4 (ab10158) were bought from Abcam. Other antibodies used were anti-β-actin AC-74 (Sigma-Aldrich, A2228) and anti-H3K27me3 (Diagenode, C15410195). Conjugate horseradish peroxidase (HRP) goat anti-rabbit (GtxRb-003-DHRPX) and goat anti-mouse (GtxMu-003-EHRPX.0.05) (Immunoreagents Inc.) were employed for immunoblotting detection.

### Cell Lines and Treatments

ATCC human U-937 and K-562 cell lines, and DSMZ human NB-4 cells, were kept in standard and unvaried atmosphere conditions (37°C in a 5% CO_2_ humidified air) employing phenol red RPMI-1640 (Euroclone) plus 2 mM L-glutamine (Gibco), 10% fetal bovine serum (FBS; Euroclone), and 100 mg/ml penicillin–streptomycin (Gibco) as a medium. A density of 2 × 10^5^/ml cells was seeded and grown in fresh medium with or without GSKJ4 at indicated times and concentrations. GSKJ4, PD98059, H89, and MG132 compounds were dissolved in dimethyl sulfoxide (DMSO), whereas PKF118-310 was prepared in H_2_O. In order to obtain the final concentrations required, a single compound was diluted in the medium, and the same amount of solvent(s) (generally less than 0.1% v/v) was employed as internal control.

### Dye Exclusion Test for Cell Proliferation Assessment

U-937 and K-562 cells (2 × 10^5^ cells/ml) were plated and treated at different times and concentrations. Afterward, 10 μl of cell suspension was diluted 1:1 in 10 μl of trypan blue (Sigma-Aldrich) and examined by optical microscope. Dead blue-stained cells were discriminated from living unstained cells for quantitative analysis. Experimental procedures were performed in triplicate, and representative results report both means and standard deviations as shown in figure.

### Cell Viability Assay

To assess the relative cell viability in reaction to specific stimuli, a density of 3 × 10^3^ cells/well in 96-well plates were seeded and treated as described in the Results section. Viable cells in each well were estimated by adding 100 μl of 5 mg/ml of 3-[4,5-dimethylthiazol-2-yl]-2,5-diphenyltetrazolium bromide (MTT solution) at the end of each experimental time point. After 3 h of incubation at 37°C, 100 μl/well of isopropanol-HCl 0.04 N (dissolving solution) was added to melt down formazan crystals. Following 30 min of incubation at room temperature on horizontal shaking, absorbance intensity was determined at 570 nm by microplate reader (Infinity 200, TECAN). All procedures were carried out at least three times, and for each data point, six replicates were performed. Representative figures show means and standard deviations.

### Cell Cycle Analysis

Cell cycle analysis was assessed as formerly described (26). In detail, cells were plated at a density of 2 × 10^5^ cells/ml, collected after stimulation, centrifuged (5 min at 400 × g) and suspended in 500 μl 1 × phosphate buffered saline (PBS), in which NP-40 (0.1%), sodium citrate (0.1%), and PI (50 mg/ml) were previously added. After incubation at room temperature in the dark for 30 min, samples were analyzed for cell cycle distribution using FACS-Calibur (BD Bioscience). At least 50 K events per sample were acquired using CellQuest software (BD Bioscience), whereas ModFit LT V3 software (Verity) was employed to determine the relative percentage number of each cell cycle phase. Biological replicates have been performed in triplicate.

### Histone Extraction

U-937 cells were collected at the end of each experimental procedure. Next, cells were resuspended twice in PBS and centrifuged again before adding TEB buffer [PBS with 0.5% Triton X-100 (v/v), 0.02% NaN3 (w/v), and 2 mM phenylmethylsulfonyl fluoride (PMSF)] with a ratio of 10^7^ cells/ml. After 10 min on ice, samples were centrifuged, and supernatants were discarded. A similar step, without incubation, was repeated using half TEB volume. Finally, pellet was dissolved in 0.2 N HCl overnight at 4°C, and the supernatant was harvested after additional centrifugation. Bradford assay was employed to define the relative protein content/sample. All spin-down cycles were carried out at 400 × g for 10 min at 4°C.

### Protein Extraction and Immunodetection

Cells were resuspended in 3–5 volumes of RIPA buffer, containing NP-40 (1%), sodium deoxycholate (0.5%), sodium dodecyl sulfate (SDS) (0.1%), aprotinin (10 μg/ml), leupeptin (1 mM), and PMSF (1 mM) and incubated on ice for 1 h. After centrifugation (18,000 × g for 15 min at 4°C), supernatant was collected and protein concentration was determined by Bradford method. Laemmli buffer 4 × was added to each sample before boiling at 95°C for 5 min. Typically, a quantity of 20–40 μg of total extracts was applied to polyacrylamide gel (Bio Rad Laboratories); thereafter, proteins were divided by weight in SDS–polyacrylamide gel electrophoresis (PAGE) and moved on nitrocellulose membrane (Sigma-Aldrich) using Mini Trans-Blot BioRad (Bio Rad Laboratories). In order to fill uncovered spots, obtained films were incubated in nonfat milk (5% w/v) and then blotted overnight with primary antibody according to the experimental procedures. The next day, HRP-conjugated goat anti-rabbit or anti-mouse was used to detect protein–antibody complexes. Each incubation was preceded and followed by 5 min wash with TBS Tween-20 (Thermo Fisher Scientific) for three times. Finally, nitrocellulose membranes were detected by standard chemical luminescence method ECL (Euroclone). Immunoblotting signals were captured using Chemi-Doc XRS (Bio-Rad Laboratories) and quantitatively analyzed by ImageJ (NIH, Bethesda).

### RNA Extraction, RT-PCR, and Real-Time PCR

With the aim of preventing RNA degradations and contaminations, RNase-free materials and solutions, prepared with diethyl pyrocarbonate (DEPC) water (Sigma-Aldrich), were used for RNA extraction. Total RNA was pulled out by Trizol (Invitrogen-Life Technologies) and successively reverse transcribed using SuperScript VILO kit (Invitrogen). Relative mRNA levels of specific genes of interest were determined by RT-PCR amplification made with iQ SYBR GREEN Supermix (Bio-Rad Laboratories). Glyceraldehyde 3-phosphate dehydrogenase (GAPDH) was used as a housekeeping gene to normalize data through ΔΔCT method. CREB and GAPDH primer sequences are reported as follows: CREB-forward: 5′-CACCTGCCATCACCACTGTAA-3′; CREB-reverse: 5′-GCTGCATTGGTCATGGTTAATGT-3′; GAPDH-forward: 5′-GGAGTCAACGGATTTGGT-3′; GAPDH-reverse: 5′-CTTCCCGTTCTCAGCCTT-3′.

### MiRNA Real-Time PCR

Following RNA extraction, the miRNA fraction was converted into cDNA using miScript Reverse Transcription Kit (Qiagen). In detail, 1 μg of RNA was incubated with 1 × Buffer, 1 × miScript RT, and DEPC-H_2_O for 60 min at 37°C and then 5 min at 95°C. Subsequently, miRNA Real-Time PCR was performed with QuantiTect SYBR Green PCR Kit (Qiagen) using 75 ng of cDNA in the presence of 1 × QuantiTect SYBR Green PCR Master Mix, miScript Universal Primer, and primer specific for miR-34b (Qiagen); RNU6b (Qiagen) specific primer was used to normalize data. The thermal protocol was as follows: 95°C for 15 min plus 35 cycles at 94°C for 15 s, 55°C for 30 s, and 70°C for 30 s.

### Statistical Analysis

Statistical analyses were performed for all biological and technical replicates. In details, Student's *t*-test was applied for the purpose of defining significant differences in the average between two distinct experimental groups, whereas analysis of variance (ANOVA) was used to test differences among more than two clusters. *P*-values of <0.05 were assumed as significant and graphically indicated with asterisks.

## Results

### GSKJ4 Inhibits Acute Myeloid Leukemia Cell Proliferation

To test the possible GSKJ4-mediated consequences on CREB expression and function in AML, U-937 cell line was used as a widely known representative model of human leukemia cells (27–29). Nevertheless, specific key experiments were also performed in other leukemia cells. In agreement with previous findings, we used GSKJ4 in a range from 1 to 10 μM (16–25).

Over the past few years, GSKJ4 has been reported to exert antiproliferative properties in different tumor cell types but not in normal cells, where no toxicity has been observed (16–23). More recently, Li et al. (25) have highlighted the therapeutic potential of GSKJ4 also for AML. According to the latter findings, we also provided evidence in which GSKJ4 causes growth inhibition in myeloid leukemia cells (30).

**Figure 1:**
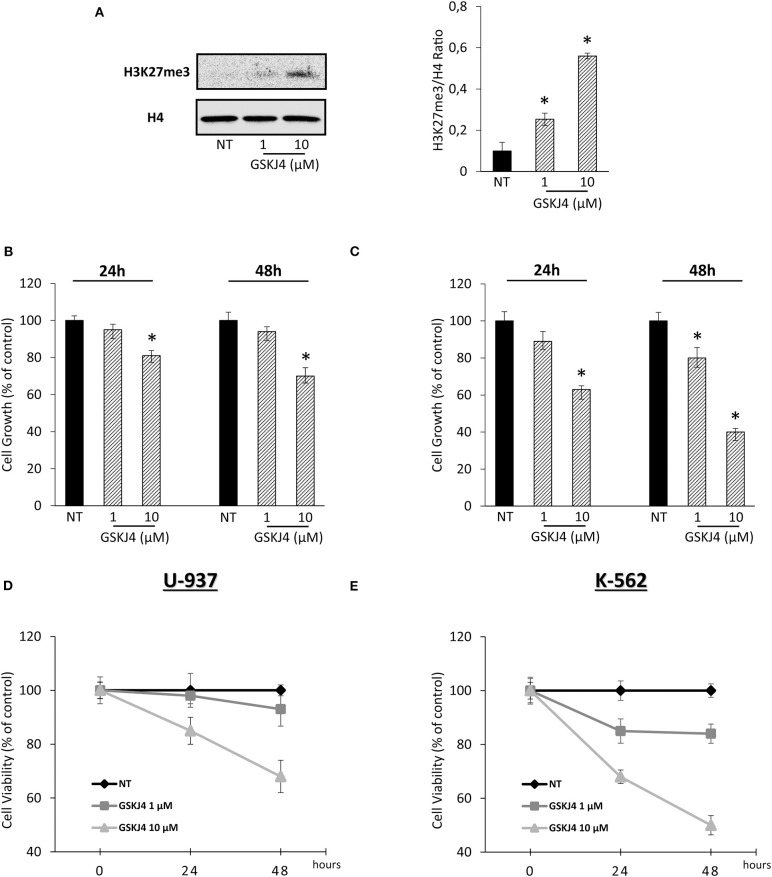
Effects of GSKJ4 on H3K27me3 status in U-937 cells and cell viability/growth in U-937 and K-562. **(A)** H3K27 trimethylation levels were measured in U-937 cells treated or not (NT) with 1 and 10 μM of GSKJ4 for 48 h. U-937 **(B)** and K-562 **(C)** cells were cultured with or without (NT) 1 and 10 μM of GSKJ4 for 24 and 48 h. Later, 3-[4,5-dimethylthiazol-2-yl]-2,5-diphenyltetrazolium bromide (MTT) assays were performed in order to determine the relative cell viability amount. U-937 **(D)** and K-562 **(E)** cell number were recorded after treatment with 1 and 10 μM of GSKJ4 at 24 and 48 h. Cell viability and cell growth data were indicated in % of control. Mean and standard deviation (SD) values of at least three repeated experiments were reported. *P < 0.05 by unpaired *t*-test.

In order to confirm our previous experimental setting, we verified the GSKJ4-mediated effects on both H3K27me3 status and leukemia cell viability. Acting as JMJD3/UTX inhibitor, Figure 1A shows that GSKJ4 increases H3K27me3. Simultaneously, analysis of cell growth and viability in U-937 and K-562 revealed an antiproliferative outcome in a dose- and time-dependent manner (Figures 1B–E).

### GSKJ4 Downregulates cAMP Response Element-Binding Protein Level in Acute Myeloid Leukemia Cells

**Figure 2:**
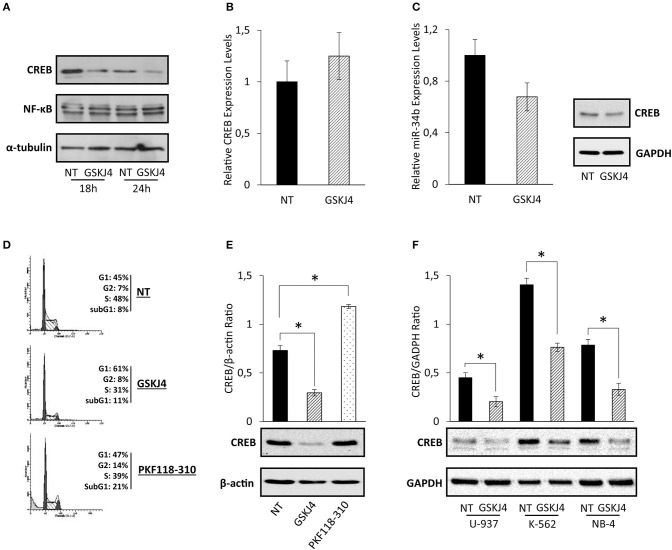
GSKJ4-mediated effects on cAMP response element-binding (CREB) protein, CREB mRNA, and miR-34b: reliability and specificity perspectives. **(A)** CREB and NF-κB protein levels were defined by immunoblotting after 18 and 24 h of treatment with 10 μM GSKJ4. CREB **(B)** and miR-34b **(C)** mRNA expression levels were assessed by RT-PCR after 24 h of 10 μM GSKJ4 treatment. GAPDH was used as a housekeeping gene, whereas differences were calculated by ΔΔCt method. **(D)** Representative cell cycle analysis of propidium iodide (PI)-stained cells achieved after 24 h of 10 μM GSKJ4 and 0.25 μM PKF118-310 treatment were reported. **(E)** CREB protein levels and CREB/β-actin Ratio, obtained in response to the same experimental setting illustrated in **(D)**, were also displayed. **(F)** U-937, K-562, and NB-4 cells were kept under GSKJ4 treatment for 24 h. Later, CREB protein levels were analyzed by Western blotting. Data from three biological replicates were used to calculate average and SD values shown in the densitometric histogram plot. *P < 0.05 by ANOVA and unpaired *t*-test.

Assuming that CREB has been identified as one of the most relevant transcription factors in AML, driving both growth and survival (5, 9), we supposed that CREB transcription factor might be involved in the antiproliferative action made by GSKJ4 in AML cells. To address our hypothesis, firstly we looked at GSKJ4-mediated effects on CREB expression. To this purpose, U-937 cells were exposed and not to GSKJ4, then CREB protein and RNA levels were evaluated by Western blotting and RT-PCR, respectively. Interestingly, as shown in Figure 2A, CREB protein level evidently decreased after 18 and 24 h following GSKJ4 treatment, whereas CREB mRNA level was not influenced (Figure 2B).

MicroRNA-34b (miR-34b) regulates CREB protein expression in myeloid cells, where, supporting CREB overexpression, it is generally downregulated and hyper-methylated (31, 32). In order to evaluate whether miR-34b was involved in GSKJ4-induced CREB protein downregulation, we determined the relative expression levels following GSKJ4 stimulation. Intriguingly, Figure 2C shows that GSKJ4 further reduced miR-34b levels in U-937 cells, excluding this translational control from GSKJ4-mediated CREB protein regulation.

With the purpose of increasing the consistency of our findings, we also looked at GSKJ4 impact on CREB protein levels in other leukemia cell models, such as K-562 and NB-4. Experimental results revealed that GSKJ4 induces CREB protein downregulation in all cell lines tested (Figure 2F). In addition, analyses of other transcription factors in response to GSKJ4, as well as of other histone demethylase inhibitors on CREB protein levels, suggest discrete and not widespread GSKJ4-mediated effects, reinforcing the specificity of our results. Indeed, NF-κB protein levels appeared unchanged upon GSKJ4 treatment (Figure 2A), whereas PKF118-310 demethylase inhibitor (33), despite being more effective compared to GSKJ4 in inducing cell cycle and death variations (Figure 2D), did not elicit CREB protein downregulation in U-937 cells (Figure 2E).

### GSKJ4 Affects cAMP Response Element-Binding Protein Stability via Ubiquitin/Proteasome System

**Figure 3:**
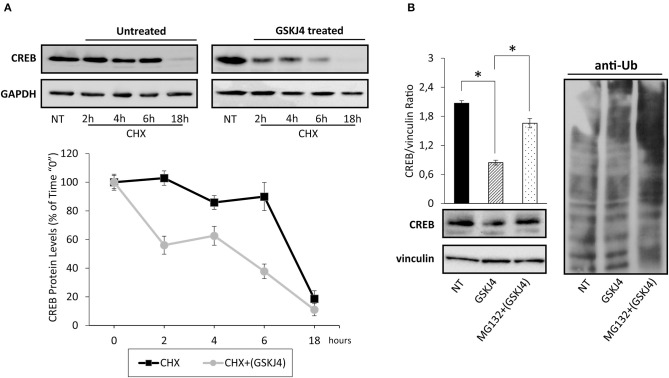
Assessment of cAMP response element-binding (CREB) stability and ubiquitin/proteasome involvement in GSKJ4-mediated CREB downregulation in U-937 cells. **(A)** Cells were treated for up 18 h with or without (NT) 10 μM GSKJ4 and grown in the presence of 20 μg/ml of protein synthesis inhibitor cycloheximide (CHX). Thereafter, CREB protein levels were determined by Western blotting (upper part) after 2, 4, 6, and 18 h of treatment. For each time point, the relative CREB/vinculin Ratio of three unrelated blot images were quantified by ImageJ software and reported in graph as average and SD measure (bottom part). **(B)** Proteasome inhibitor MG132 at 10 μM was administered, or not (NT), for 18 h to U-937 cells kept in the presence of 10 μM GSKJ4. CREB protein levels and the ubiquitin pattern were evaluated through Western blotting analysis in response to the above stimuli. Densitometric analyses of CREB/vinculin Ratio of three independent experiments were shown in chart and represented as average and SD values. *P < 0.05 by ANOVA and unpaired *t*-test.

To test if GSKJ4 might decrease CREB protein expression levels affecting its stability, we firstly estimated CREB protein half-life in untreated (control) and GSKJ4-treated U-937 cells by the broadly used protein synthesis inhibitor cycloheximide (CHX) (34). Hence, U-937 cells were treated or not with GSKJ4 and cocultured in the presence of CHX for 18 h, afterward, CREB protein levels at different time points were analyzed. Interestingly, Figure 3A shows that CREB protein levels were clearly decreased already after 2 h of CHX exposure in GSKJ4-treated group, whereas a significant reduction in the CREB abundance was evident only after 18 h of exposure to CHX in the untreated group, suggesting that GSKJ4 compound effectively influences CREB stability.

To investigate the mechanisms by which GSKJ4 decreases CREB protein stability, we investigated the impact of MG132 proteasome inhibitor on CREB abundance in response to GSKJ4 treatment. Therefore, U-937 cells were kept under proteasome impairment and treated with or without GSKJ4 for 18 h. Subsequently, we aimed to evaluate both CREB and anti-ubiquitin expression levels, the latter for monitoring the effectiveness of MG132 as proteasome inhibitor. Figure 3B clearly shows that the presence of MG132 increased the poly-ubiquitin signal and, more interestingly, it largely rescued the GSKJ4-induced CREB downregulation, suggesting the involvement of the ubiquitin/proteasome pathway in CREB protein decrease in response to GSKJ4.

Taken together, these findings indicate that the GSKJ4 treatment causes a decrease of CREB protein in U-937 cells, affecting its stability *via* ubiquitin/proteasome system.

### GSKJ4 Rapidly Induces cAMP Response Element-Binding Phosphorylation at Residue Ser133 That Precedes the cAMP Response Element-Binding Protein Downregulation

**Figure 4:**
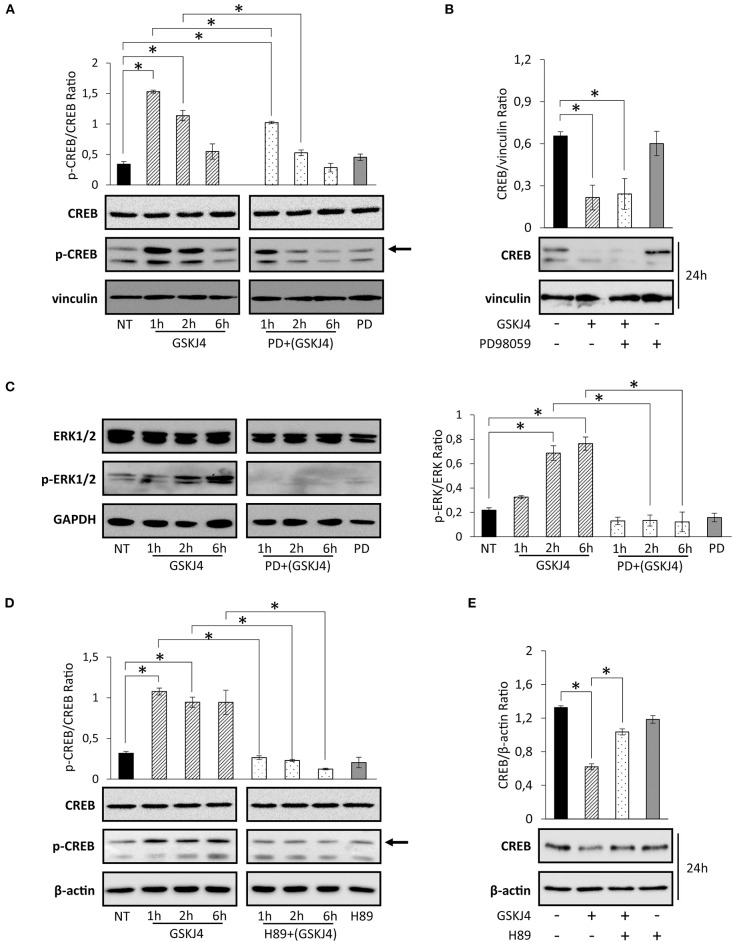
Effects of PD98059 and H89 on GSKJ4-mediated Ser133 phosphorylation induction and cAMP response element-binding (CREB) protein decrease. U-937 cells were treated up to 24 h with 10 μM GSKJ4 in the presence or in the absence of 10 μM PD98059 or 20 μM of H89. Immunoblotting analyses of CREB and phospho-Ser133-CREB were performed after 2, 4, and 6 h of treatment with GSKJ4 alone and in combination with PD98059 **(A)** or H89 **(D)**. Untreated (NT), PD98059 and H89 lines refer to 2 h of treatment. **(C)** ERK1/2 and phospho-ERK1/2 levels were evaluated in the same experimental setting described for **(A)**. CREB protein levels and CREB/housekeeping protein Ratio were determined by Western blotting in response to 24 h of treatment with GSKJ4 alone and in combination with PD98059 **(B)** or H89 **(E)**, respectively. All densitometric analyses shown in this figure were calculated from three distinct experiments. *P < 0.05 by ANOVA and unpaired *t*-test.

Activation of transcription factors usually requires posttranslational modifications such as phosphorylation (35, 36). Moreover, in many of them, phosphorylation and dephosphorylation processes regulate stability and degradation, too (37, 38). Regarding CREB transcription factor, it is largely known that phosphorylation at Ser133 residue might modulate both activation and protein stability (39–44). To extend and further investigate the impact of GSKJ4 on CREB function, we investigated Ser133 phosphorylation and CREB protein levels in response to GSKJ4 treatment. To corroborate our study, we treated U-937 cells with GSKJ4 at different times, and then we evaluated the p-Ser133-CREB and CREB protein levels. Interestingly, as shown in Figure 4A (left panel), we found that CREB phosphorylation strongly increased in response to GSKJ4 already after 1 h, without relevant changes in CREB total levels up to 6 h, suggesting a very early CREB activation that seems to precede CREB protein downregulation.

Overall, the above results indicate that GSKJ4 greatly impacts p-Ser133-CREB protein levels, suggesting that this mechanism might mediate the GSKJ4-induced CREB downregulation.

### Protein Kinase A Activity, and Not Extracellular Signal-Regulated Kinase 1/2 Activity, Is Mainly Required in GSKJ4-Induced cAMP Response Element-Binding Protein Downregulation

Among the others kinases, ERK1/2 and PKA play a key role in CREB phosphorylation at Ser133 residue (45–48). To test the possible involvement of ERK1/2 pathway on GSKJ4-mediated Ser133 phosphorylation induction and CREB protein decrease, we treated U-937 cells with GSKJ4 up to 24 h in presence or in absence of the upstream ERK1/2 inhibitor PD98059. Later, we evaluated the p-ERK1/2, p-Ser133-CREB, ERK1/2, and CREB protein expression levels. Notably, as previously described, cell exposure to GSKJ4 provokes a rapid increase of p-Ser133-CREB (Figure 4A, left panel) that was partially prevented by pretreatment with PD98059 compound (Figure 4A, right panel). Strikingly, the GSKJ4-induced CREB protein downregulation was not counteracted at all by the ERK1/2 inhibitor PD98059 (Figure 4B). Indeed, as shown in Figure 4C, PD98059 was able to totally abrogate ERK1/2 activation in response to GSKJ4 compound (Figure 4C). Collectively, the above findings indicate that GSKJ4-mediated phosphorylation on serine 133 residue is partly dependent on ERK1/2 activity, whereas CREB protein downregulation is probably affected by other independent mechanisms.

In order to define the potential PKA implication on both GSKJ4-mediated Ser133 phosphorylation and CREB protein downregulation, GSKJ4-treated U-937 cells were cultured both with or without PKA inhibitor H89, and subsequently, total and phosphorylated CREB protein expression levels were analyzed. Remarkably, Figures 4D,E show that pretreatment of U-937 cells with the H89 inhibitor results in both a strong reduction of the p-Ser133-CREB phosphorylation and an almost complete prevention of CREB protein downregulation in response to GSKJ4 compound. Altogether, the above findings indicate that both CREB phosphorylation on Ser133 residue and CREB downregulation are largely dependent on PKA activity.

## Discussion

Despite the intensive combination of chemotherapy and stem cell transplantation, AML still remains very difficult to treat (3, 49). It is known that altered functions, such as transcription factors mutation and/or dysregulated expression are deeply involved in leukemogenesis (4). Although therapeutic targeting of transcription factors is still considered challenging, recent evidence is showing that transcription factors can be targeted for cancer care (50–53). Remarkably, targeting the activity of CREB transcription factor appears very promising for the treatment of leukemia in a huge number of preclinical studies (4, 12, 54, 55).

Here, we report that the small epigenetic compound GSKJ4 significantly downregulates CREB protein levels in leukemic cells. Since GSKJ4 treatment causes a strong decrease of CREB protein levels, but not a concurrent reduction of CREB mRNA expression levels, intricate posttranslational mechanisms are supposed to be involved. To further corroborate our hypothesis, the evaluation of GSKJ4-mediated effects on miR-34b, one of the main controllers in CREB translation, reveals no engagement in its downregulation. Contextually, by CHX assay, we provide evidence that CREB protein stability decreases in response to GSKJ4 exposure and that proteasome inhibition largely counteracts the GSKJ4-induced CREB downregulation. In addition, we also describe a rapid CREB phosphorylation at Ser133 residue that seems to precede CREB protein decrease in response to GSKJ4 treatment. From a mechanistic point of view, PKA impairment almost completely prevents both the GSKJ4-induced p-Ser133-CREB and CREB protein downregulation.

Notably, targeting CREB has been recently proposed as an effective therapeutic approach in AML. CREB recognized more than thousands of CRE consensus *loci* on the DNA. Upon phosphorylation on Ser133, CREB, together with CBP coactivator, triggers the transcription of CREB-driven genes, which in turn regulate cell proliferation, survival, and signal transduction. Interestingly, Mitton et al. (12) provided evidence that a CBP–CREB interaction blocker disrupts CREB-driven gene expression, causing cell cycle arrest and apoptosis of AML cells. Subsequently, the same group, by structure–activity relationship (SAR) tools, demonstrated that niclosamide, a widely used uncoupled oxidative phosphorylation compound, targets CREB and, preventing its activation, inhibits CREB-driven gene expression and results in cell cycle arrest and apoptosis in AML cells (54, 55).

Our results, showing that the epigenetic compound GSKJ4 causes both CREB protein decrease and inhibition of proliferation in AML cells, are fully in agreement with the above findings. However, we do know that our findings are quite descriptive, thus correlative and more exhaustive experiments are needed to demonstrate the relationship between the antiproliferative effects and CREB decrease in response to GSKJ4 in AML cells. We are going to address this issue as a future scientific focus. By the way, our study clearly demonstrates that GSKJ4 downregulates CREB protein in AML cells, and this is undoubtedly relevant *per sè*.

As JMJD3 and UTX demethylase inhibitor, we have shown that GSKJ4 increases H3K27me3 status in AML cells. Moreover, GSKJ4 has recently been shown to counteract cell proliferation in different tumor types, including AML (16–25, 30).

In the current study, we describe that the GSKJ4 compound, according to previous findings, acts as a small molecule inhibitor against the proliferation of myeloid leukemia cells and elicits a rapid CREB protein downregulation, suggesting that GSKJ4 might exert anticancer properties by inhibiting demethylases JMJD3 and UTX and affecting CREB function. Changes in H3K27me2/3 global level might influence the chromatin configuration and affect the accessibility of CREB transcription factor, making it more susceptible to cellular activities such as kinases, ubiquitin ligases, and proteases. Moreover, possible GSKJ4-mediated effects on other enzymatic functions, maybe involved in CREB activation/stability, cannot be excluded. In addition, methylation of non-histone proteins has recently been demonstrated to impact diverse cellular functions. Notably, many of such non-histone targets include transcription factors, such as CREB (56).

Here we report that upon GSKJ4 treatment, an early phosphorylation of CREB at the Ser133 residue occurs and precedes the proteasome-mediated CREB protein downregulation.

It is largely known that PKA, as well as other kinases such as ERK1/2, can phosphorylate CREB protein at serine 133 residue (45–48). Moreover, phosphorylation at Ser133 is also involved in CREB protein stability (40, 41, 44). Importantly, our findings, indicating that PKA inhibition almost completely prevents both GSKJ4-induced p-Ser133-CREB phosphorylation and CREB protein decrease, are consistent with the above evidence. Based on all these statements, the molecular mechanisms underlying the effects of GSKJ4 on CREB pathway in AML cells are still preliminary and, therefore, they need to be further investigated.

Here we describe that exogenous addition of GSKJ4 epigenetic compound triggers proteasome-mediated CREB protein downregulation *via* a PKA-dependent manner.

CREB is a key regulator of the growth and survival of AML cells that is emerging as a very attractive potential drug target for AML (4, 5, 12).

Collectively, our study identifies the small-molecule GSKJ4 as a valuable potential agent capable of modulating CREB function, encouraging the design of further GSKJ4-based studies for innovative therapeutic approach in AML care.

## Data Availability Statement

The datasets generated for this study are available on request to the corresponding author.

## Author Contributions

MI performed immunoblotting experiments and participated in the development of the current study. MC executed mRNA analyses and contributed to draft the manuscript. ASa and AR carried out MTT and cell counting assays. AN and LA took an active part in the drafting process of this paper. LS made flow cytometry-based data and the statistical analysis. ASp supervised the final text layout. SN conceived and designed the research project, directing both the experimental strategies and the draft of the manuscript. Reading the present article, the authors approve its contents.

## Conflict of Interest

The authors declare that the research was conducted in the absence of any commercial or financial relationships that could be construed as a potential conflict of interest.
